# Three dimensional quantitative study of soft tissue changes in nasolabial folds after orthodontic treatment in female adults

**DOI:** 10.1186/s12903-023-02733-5

**Published:** 2023-01-19

**Authors:** Qin Zhou, Jie Gao, Donghui Guo, Haolin Zhang, Xu Zhang, Wen Qin, Zuolin Jin

**Affiliations:** 1 Key Laboratory of Shaanxi Province for Craniofacial Precision Medicine Research, College of Stomatology, Xi’an Jiaotong University, No. 98 XiWu Road, Xi’an, 710004 Shaanxi China; 2 Department of Orthodontics, College of Stomatology, Xi’an Jiaotong University, No. 98 XiWu Road, Xi’an, 710004 Shaanxi China; 3 State Key Laboratory of Military Stomatology & National Clinical Research Center for Oral Diseases & Shaanxi Clinical Research Center for Oral Diseases, Department of Orthodontics, School of Stomatology, The Fourth Military Medical University, No. 169 Changle West Road, Xi’an, 710032 Shaanxi China; 4 Department of Stomatology, The PLA Hong Kong Garrison Hospital, Hong Kong, 999077 China

**Keywords:** Female adults, Extraction, Non-extraction, Facial soft tissue, Facial aesthetics, Nasolabial folds, 3dMD

## Abstract

**Background:**

With the popularity of medical aesthetic programs, some female adults who will or are undergoing orthodontic treatment often wonder whether orthodontic treatment has adverse effects on the nasolabial folds (NLFs). The aims of the study were to investigate any potential changes in the NLFs and associated peripheral soft tissues after orthodontic treatment of female adults.

**Methods:**

This study compared changes in the NLFs and peripheral soft tissues in female adults undergoing orthodontic treatment using the 3dMD Face system (3dMD, Atlanta, Ga). A total of 52 adult female patient cases (24 teeth extraction, 28 non-teeth extraction) were included to evaluate the effects of different orthodontic treatment regimens on the NLFs and peripheral soft tissues.

**Results:**

In the NLFs area, the landmarks of the extraction group were all significantly negatively changed (*P* < 0.001; the NLF2s average value was − 0.72 mm), and the upper and middle parts of the landmarks were negatively changed in the non-extraction group (*P* < 0.05; the NLF2s average value was − 0.22 mm). Compared to the non-extraction group, the negative changes in the extraction group were more pronounced (*P* < 0.005). In the lip region, all landmarks in the extraction group were negative changes (*P* < 0.05; upper lip (ULP) = − 0.93 mm, lower lip (LLP) = − 1.46 mm), and most landmarks in the non-extraction group were positive changes (*P* < 0.01; ULP = 0.55 mm). In the cheek area, the left and right buccal of the extraction and non-extraction groups were all negatively changed (*P* < 0.05), and there was no significant difference between the two groups.

**Conclusion:**

After orthodontic treatment, the NLFs showed negative changes, which were more obvious in the extraction group. The lip soft tissue had a negative change in the extraction group and a positive change in the non-extraction group, indicating that orthodontic treatment affected the soft tissue around the nasolabial sulcus, and that tooth extraction would lead to more negative changes.

## Background

An increasing number of adult female patients are seeking orthodontic treatment, with an improvement in their facial aesthetics being one of their primary goals [1, 2]. Orthodontic treatment will not only change the maxillofacial hard tissues but also the corresponding soft tissues [3]. After orthodontic treatment, there is an increasing concern about changes in the soft tissues of the face compared to the hard tissues [4]. Studies have shown that the shape of the bone is relatively stable over a period of 2–3 years [5]. Therefore, any changes in the face after orthodontic treatment may be caused mainly by changes in the soft tissues, which needs to be proven by more rigorous research. Previous studies have shown that orthodontic treatment may cause changes in the soft tissues such as the buccal region, cheek region and zygomatic arch area, regardless of whether extraction or non-extraction occurred [3]. Nasolabial folds (NLFs) are located between the lip and the cheeks and extend from the nose to the mouth [6]. A change in the NLFs can have an intuitive effect on facial youth, with the deepening of the NLFs being the most obvious feature of facial aging [7, 8]. In clinical work, we sometimes find that the NLFs become more pronounced in some patients after tooth extraction; however, there are few reports on the morphological changes of NLFs after orthodontic treatment.

In the past, two-dimensional methods such as X-ray or photography [9] were mostly used in facial soft tissue research [10]. However, these studies did not provide a realistic and accurate three-dimensional view of the patient’s facial morphology. Currently, commonly used 3D imaging systems include laser scanning, 3D photography and cone-beam computed tomography (CBCT), all of which provide a more realistic and comprehensive view of the patient’s face [11]. The image from laser scanning is not accurate enough, and in addition takes a long time to capture. When capturing facial information, errors will occur due to the movement of the subject, and the high-frequency laser beams will cause discomfort. CBCT has high accuracy, but it generates radiation, which has a negative impact on the human body. The 3dMD face system (3dMD, Atlanta, Ga) can acquire 3D images and its reliability has been extensively proven [12–16]. In recent years, the later face system has been widely used in maxillofacial soft tissue research. However, to date little data have been published on potential changes in the NLFs and their associated peripheral soft tissues after orthodontic treatments or differences in changes in NLFs caused by extractive and non-extractive orthodontic therapy.

In the present study, the 3dMD stereo photography system was used to analyze changing trends in NLFs and the surrounding soft tissue after orthodontic treatment in female adults, and to compare the effects of tooth extraction and non-extraction treatment on the NLFs.

## Methods

### Subject selection

The sample was comprised of a cohort of 52 patients referred to the Department of Orthodontics, School of Stomatology, the Fourth Military Medical University, Xi’an, Shaanxi, China. Of these, 28 had non-extraction treatment (non-extraction group, aged 24.83 ± 3.46 years), and 24 extraction treatment (extraction group, aged 25.09 ± 4.04 years). The inclusion criteria for the two groups were: (1) Han-Chinese women, with ages ranging from 18 to 35 years; (2) Having complete pretreatment and posttreatment 3dMD images; (3) Class I or II malocclusion (0 < ANB < 6°); (4) Extraction groups were treated with extraction of 4 first or second premolars; (5) Body Mass Index (BMI) between 18.5 and 24 kg/m^2^. The exclusion criteria were: (1) A history of previous orthodontic or orthopedic treatment; (2) Facial asymmetry (> 5 mm of soft-tissue menton (Me’) deviation) or deformity; (3) Maxillofacial trauma and surgery history; (4) Injection or implantation surgery to change facial shape, such as hyaluronic acid or botulinum toxin injections, history of prosthesis implantation, etc.; (5) Temporomandibular joint disorders with obvious clinical symptoms; (6) Pregnancy; (7) Systemic disease that could affect the dentition, poor cooperation and/or inadequate oral hygiene.

This study followed the guidelines of the Declaration of Helsinki (2013). Ethical approval was obtained from the Fourth Military Medical University, Xi’an, Shaanxi Province, China (IRB-REV-2,021,044) and all participants gave approval.

### Extraction of premolars

The main reasons for premolars extraction were protrusion, crowding, and both. For patients with tooth extraction, the first or s premolars were extracted according to anterior crowding and malocclusion classification. When anterior crowding was greater than 8 mm and the molar relationship was Class I malocclusion, the upper and lower 4 first premolars need to be removed. When anterior crowding was less than 8 mm and the molar relationship was Class I malocclusion, the upper and lower 4 s premolars were extracted. When the molar relationship was Class II malocclusion, 2 first premolars should be extracted from the maxillary and 2 s premolars should be extracted from the mandibular. In addition, when patients were protrusion, priority was given to the removal of 4 first premolars. If any premolars have caries, pulpitis or root dysplasia, these premolars should be preferentially removed. In all patients, the extraction time was 1 week before bracket bonding.

### Orthodontic process

All patients were treated with a 0.022 × 0.028 inch straight-archwire appliance and labial bracket system, and second molars were included in the orthodontic treatment. The anterior teeth were retracted by sliding mechanics in extraction patients. When moving the front teeth of an extraction patient backwards, they could choose different anchors to help move the teeth as required. The anchorage devices used in the extraction patients were micro-implants, transpalatal arch (TPA); no external anchorage devices were used. All extracted patients had four orthodontic teeth removed one week prior to upper bracket bonding, and the lower brackets were bonded 1 month after upper bracket bonding.

### 3dMD image acquisition

**Fig. 1:**
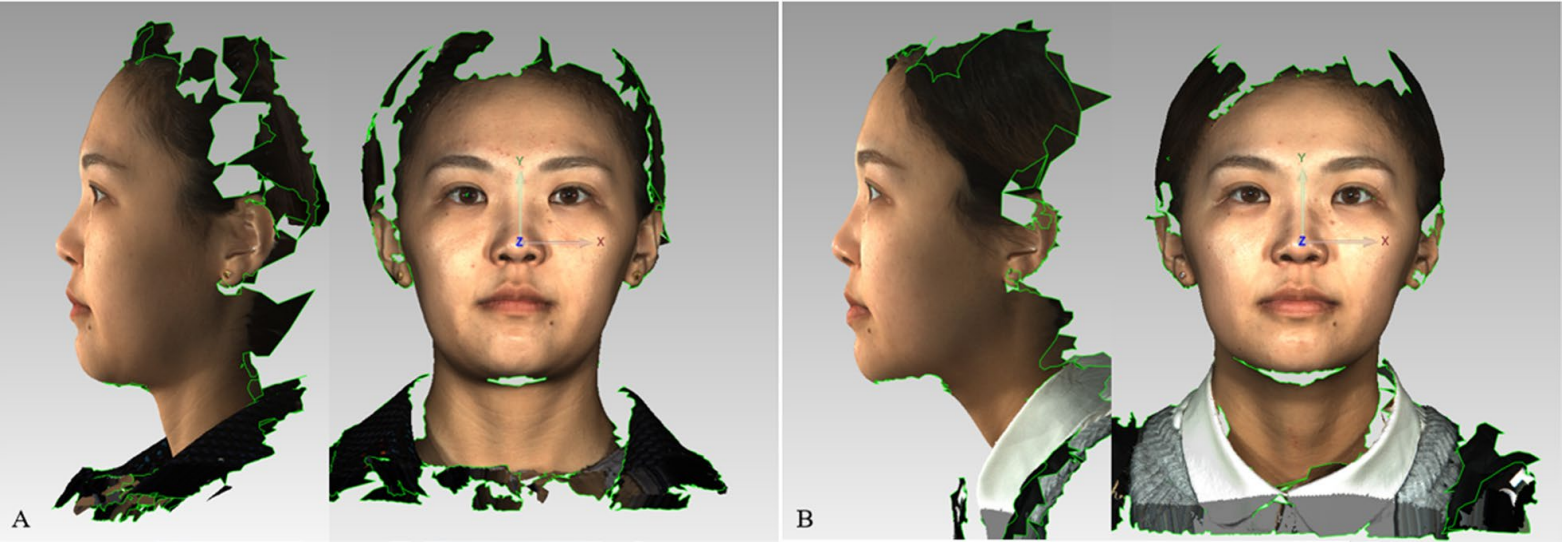
Generation of 3dMD images. **A**, 3dMD images of subjects in the T0 period; **B**, 3dMD images of subjects in the T1 period

Orthodontic patients had 3dMD facial images taken before treatment (T0) and after bracket removal at the end of treatment (T1). The acquisition of 3D data was performed by the same operator. During the shooting, the patient sat upright in the center of the instrument, with their head in a natural position, the face having no micro-expression, teeth in a central occlusion state, and the lips in a static state. No hat, hair or other ornaments covering the face. Then, the lighting and chair position were adjusted so that the patient’s head was on the screen. In this optimal position, the patient remained motionless and a camera image was captured every 1.5 ms, and used to generate the 3dMD images (Fig. 1). The 3dMD images was saved in the OBJ format.

### 3dMD refinement and overlap

**Fig. 2:**
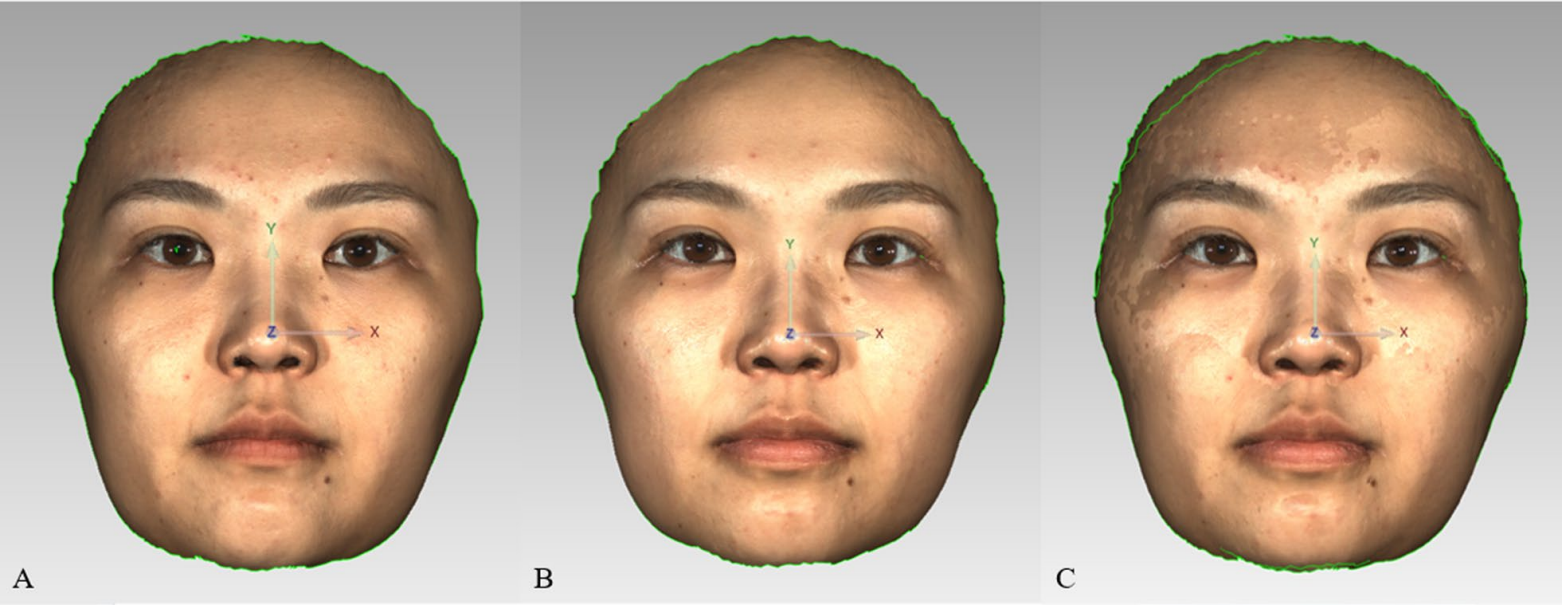
Refinement and overlap of 3dMD images.** A**, removal of the flash of the T0 facial image; **B**, removal of the flash of the T1 facial image; **C**, T0T1 overlapping image

**Fig. 3:**
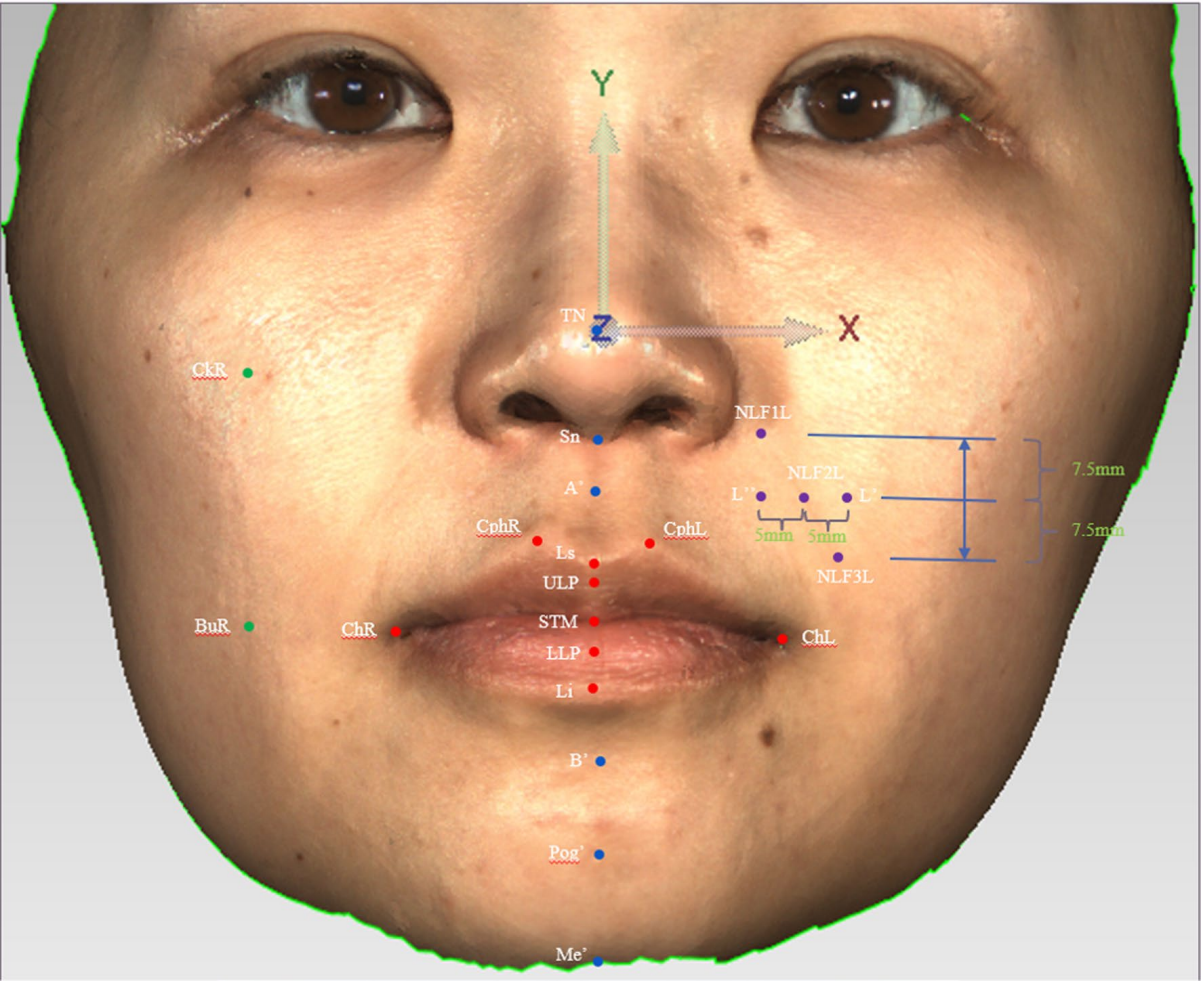
The position of landmarks

The 3dMD facial images of subjects T0 and T1 were imported into Geomagic control (Geomagic2014, Research Triangle Park, NC) software and the hair, ear, neck and clothing were digitally removed. Relatively stable reference points were selected on the forehead and the nasal root areas of patients. Overlapping of T0 as the reference image and T1 as the test image was employed to obtain the overlapped image (Fig. 2). The 3dMD facial image measurements sites are given in Fig. 3; Table 1.

**Table 1:** Landmarks of the nasolabial folds and its peripheral soft tissues

Landmarks	Definitions
*Nasolabial folds*	
NLF1L	The left upper nasolabial folds (At the level of the subnasal of the left nasolabial fold)
NLF2L	The left middle nasolabial folds (At the level of 7.5 mm vertically downward from the Subnasal of the left nasolabial fold)
NLF3L	The left lower nasolabial folds (At the level of 15 mm vertically downward from the subnasal of the left nasolabial fold)
L′	Outside of the left nasolabial folds (0.5 mm out of the NLF2L level)
L′′	Inside of the left nasolabial folds (0.5 mm inward of the NLF2L level)
NLF1R	The right upper nasolabial folds (At the level of the subnasal of the right nasolabial fold)
NLF2R	The right middle nasolabial folds (At the level of 7.5 mm vertically downward from the subnasal of the right nasolabial fold)
NLF3R	The right lower nasolabial folds (At the level of 15 mm vertically downward from the subnasal of the right nasolabial fold)
R′	Outside of the right nasolabial folds (0.5 mm out of the NLF2R level)
R′′	Inside of the right nasolabial folds (0.5 mm inward of the NLF2R level)
*Lips*	
ChL	Cheilion left (Left corner of the mouth)
ChR	Cheilion right (Right corner of the mouth)
ULP	Upper lip (Most prominent point of the upper lip)
LLP	Lower lip (Most prominent point of the lower lip)
Ls	Labrale superior (Highest point of the lower lip)
Li	Labrale inferior (Lowest point of the lower lip)
CphL	Crista philtri left
CphR	Crista philtri right
STM	Stomion (Midpoint of upper and lower mouth cracks)
*Cheeks*	
CkL	Cheek left (The intersection of the horizontal extension line passing through the midpoint of the left nasal alar and the vertical extension line passing through the left exocanthion)
CkR	Cheek right (The intersection of the horizontal extension line passing through the midpoint of the right nasal alar and the vertical extension line passing through the right exocanthion)
BuL	Buccal left (The intersection of the horizontal extension line passing through the left corner of the mouth and the vertical extension line passing through the left exocanthion)
BuR	Buccal right (The intersection of the horizontal extension line passing through the right corner of the mouth and the vertical extension line passing through the right exocanthion)
*Perioral soft tissues*	
TN	Tip of nose
Sn	Subnasale
A′	Soft-tissue A-point
B′	Soft-tissue B-point
Pog′	Soft-tissue pogonion
Me′	Soft-tissue menton

### Quantitative assessments of facial soft tissue

**Fig. 4:**
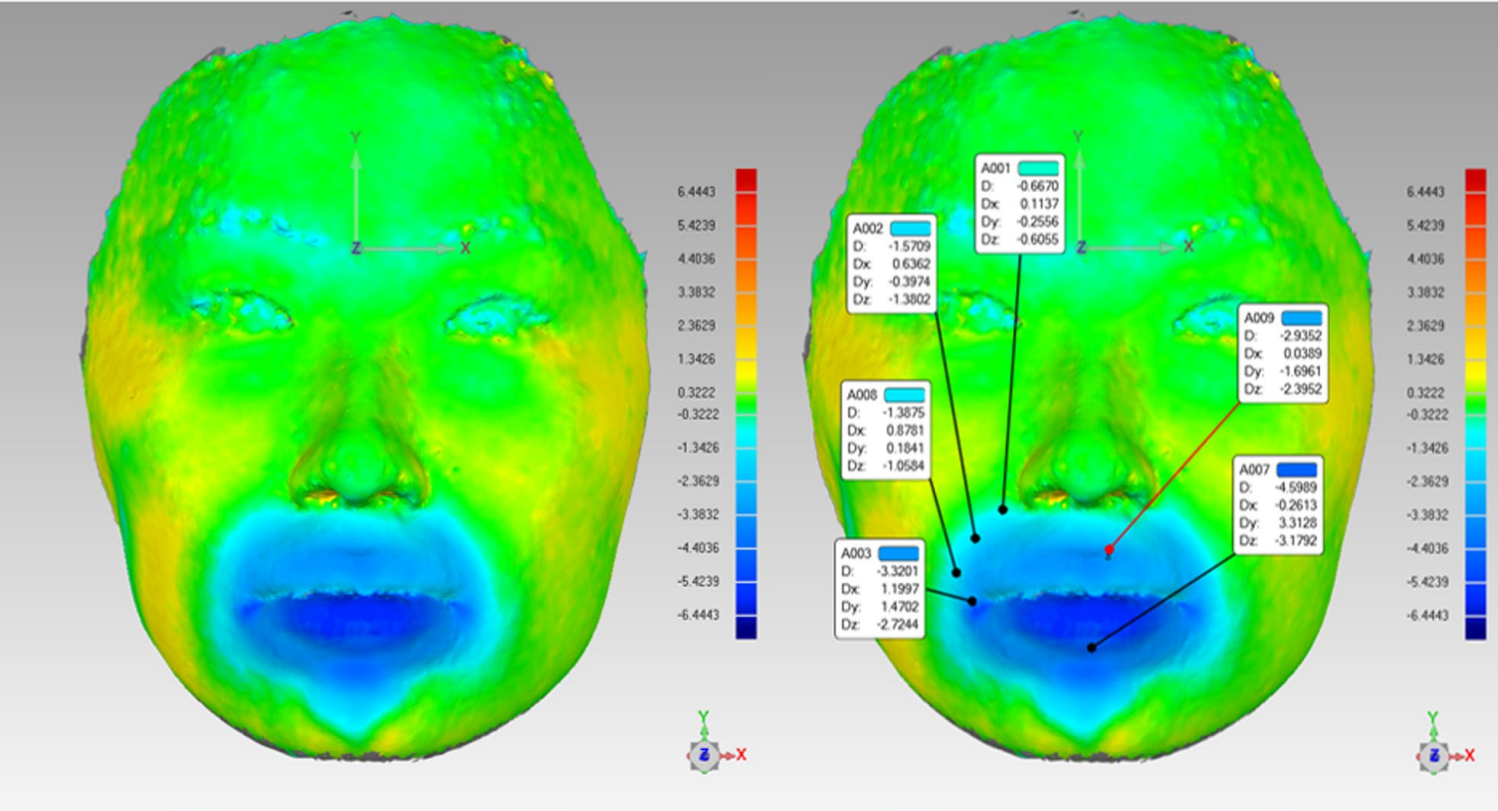
Generation of absolute color mapping and measurement of landmarks. Green indicates that the amount of soft tissue change did not exceed the tolerance level and the reading is close to zero; blue indicates that the soft tissue became flat or depressed and the reading is negative, indicating that the orthodontic treatment had caused a negative change in the soft tissue, manifesting as an inward depression; red indicates that the soft tissue became more convex and the reading was positive, indicating that the orthodontic treatment had caused a positive change in the soft tissue, manifesting as an outward protrusion

First, an absolute color mapping for the overlay was generated (Fig. 4). The distance between the T0 and T1 images was analyzed qualitatively and quantitatively using absolute color mapping. In absolute color mapping, green indicated that the amount of change in soft tissue did not exceed the tolerance level; blue indicated that the soft tissue became flat or concave with negative readings; and red indicated that the soft tissue became more convex with positive readings. Changes were recorded for 29 landmarks in 4 regions.

### Verification of repeatability and reliability

#### Verification of point selection errors

The experimental errors mainly included point selection and image overlap errors. The study was performed by the same operator and another operator. During training, the operators first selected the left upper nasolabial folds (NLF1L) point of 3dMD images for T0 for 10 patients, and then repeated the selection at one-week intervals. The X-axis value of the coordinate system of each point was obtained, and then the inter-class and intra-class coefficients was employed on the data using SPSS ver. 23.0 software. The inter-class and intra-class coefficients revealed that there was no statistical difference in the selection of points, with coefficients of 0.985 (*P* < 0.001) and 0.972 (*P* < 0.001), proving that the selection of points was reliable, consistent and unbiased.

#### Validation of overlap errors

In the present study, image overlap was performed by the same operator and another operator. Ten patients were randomly selected from 52 and overlap matching of T0 and T1 was performed at one-week intervals. The Geomagic control software automatically output the deviation level in the range of 0.20 ± 0.04 mm. Within this error range, the software automatically selected the maximum matching area. If the matched area exceeded 90%, the overlapping area was displayed using absolute color mapping, the mean value of the entire facial variation was recorded, and the inter-class and intra-class coefficients performed on the mean value of each group of images obtained. The inter-class and intra-class coefficients showed no statistical difference between the groups, with coefficients of 0.983 (*P* < 0.001) and 0.950 (*P* < 0.001), which proved that the overlap method was reliable.

### Statistical analysis

A paired *t*-test was performed on the initial ages and BMIs between the non-extractive and extractive groups. All the data were tested for a normal distribution using the Shapiro-Wilk test, and the variances uniform were tested by one-way ANOVA test.

SPSS ver. 23.0 software was used to analyze each group of data separately, and a one-sample *t*-test was employed for data that conformed to a normal distribution. The one-sample Wilcoxon’s test was used to compare data that did not conform to a normal distribution. A two independent samples *t*-test or Mann-Whitney U test were utilized to evaluate any differences between the extraction and non-extraction groups at each landmark. A paired *t*-test or Wilcoxon signed rank test was used to analyze any potential differences between different parts of the NLFs and other landmarks. Any changes in landmarks at the NLFs and surrounding soft tissues after orthodontic treatment were observed. The sample size was calculated with α = 0.05 and a power of 80%. The minimum sample size required for the present study was 13 for each group.

## Results

All 52 patients were included in the study, and there were no significant differences in the initial ages and BMIs between the groups (*P* > 0.05) (Table 2).

**Table 2:** Age, BMI distribution and comparison of the samples

	Non-extraction	Extraction	*P*-value
Age	24.83 ± 3.46	25.09 ± 4.04	0.80
T0	20.70 ± 1.55	20.10 ± 1.40	0.16
T1	20.80 ± 1.56	20.40 ± 1.27	0.35

T0, before bracket bonding; T1, at the end of the treatment

As shown in Tables 3, 4 and 5, the evaluation of the measurement results of the NLFs showed that all parts were negatively changed in the extraction group (*P* < 0.001; NLF2s average value is − 0.72 mm), indicating an inward depression of the soft tissues, suggesting that extraction orthodontics had a negative impact on the soft tissues of the NLFs. For the non-extraction group, the upper and middle parts of the NLFs were negatively changed (*P* < 0.05; NLF2s average value is − 0.22 mm), and the outer sides of the NLFs were also negatively changed (*P* < 0.05; average value is − 0.18 mm), however, for the inner side of NLFs, only the left were negatively changed (*P* < 0.05; outside of the left nasolabial folds (L′) = − 0.16 mm), indicating that non-extraction orthodontics had a negative effect on some areas of the soft tissues of the NLFs. In all measurement items of the NLFs, the changes in the extraction group were significantly different from those in the non-extraction group (*P* < 0.05), and the negative changes in the extraction group were more obvious, indicating that extraction orthodontics had a greater effect on the soft tissues of the NLFs than non-extraction orthodontics. In addition, the change degree of the middle part of the left NLFs were greater than that of the upper part in the extraction group (*P* < 0.05), which might indicate that orthodontic extraction had a greater effect on the middle part of the nasolabial sulcus.

**Table 3:** Changes in the landmarks of the nasolabial folds and their peripheral soft tissues*

Measurement	Non-extraction		Extraction	
	M ± SD	*P*-value	M ± SD	*P*-value
*Nasolabial folds*				
NLF1L	− 0.26 ± 0.33	0.000^ƚ^	− 0.59 ± 0.30	0.000^ƚ^
NLF2L	− 0.22 ± 0.38	0.004^ƚ^	− 0.72 ± 0.31	0.000^ƚ^
NLF3L	− 0.08 ± 0.44	0.345	− 0.70 ± 0.42	0.000^ƚ^
L′	− 0.16 ± 0.32	0.016^ƚ^	− 0.58 ± 0.35	0.000^ƚ^
L′′	− 0.16 ± 0.38	0.041^ƚ^	− 0.81 ± 0.45	0.000^ƚ^
NLF1R	− 0.24 ± 0.37	0.000^ƚ,ǂ^	− 0.61 ± 0.32	0.000^ƚ^
NLF2R	− 0.22 ± 0.42	0.010^ƚ^	− 0.73 ± 0.38	0.000^ƚ^
NLF3R	− 0.12 ± 0.38	0.101	− 0.66 ± 0.44	0.000^ƚ^
R′	− 0.20 ± 0.43	0.020^ƚ^	− 0.58 ± 0.39	0.000^ƚ^
R′′	− 0.16 ± 0.41	0.055	− 0.74 ± 0.53	0.000^ƚ^
*Lips*				
ChL	0.42 ± 1.54	0.157	− 1.48 ± 0.93	0.000^ƚ^
ChR	0.42 ± 1.54	0.165	− 1.43 ± 0.89	0.000^ƚ,ǂ^
ULP	0.55 ± 1.04	0.009^ƚ^	− 0.93 ± 0.78	0.000^ƚ^
LLP	0.42 ± 1.21	0.080	− 1.46 ± 0.70	0.000^ƚ^
Ls	0.49 ± 0.92	0.016^ƚ,ǂ^	− 0.63 ± 1.16	0.009^ƚ,ǂ^
Li	0.81 ± 0.97	0.000^ƚ^	− 0.96 ± 1.60	0.008^ƚ^
CphL	0.44 ± 0.78	0.009^ƚ,ǂ^	− 0.55 ± 0.96	0.010^ƚ^
CphR	0.50 ± 0.79	0.005^ƚ,ǂ^	− 0.57 ± 0.94	0.006^ƚ,ǂ^
STM	1.17 ± 0.86	0.000^ƚ,ǂ^	− 0.61 ± 1.02	0.010^ƚ,ǂ^
*Cheeks*				
CkL	− 0.13 ± 0.88	0.454	0.13 ± 0.43	0.151
CkR	− 0.12 ± 0.81	0.457	0.08 ± 0.47	0.392
BuL	− 0.22 ± 0.52	0.034^ƚ^	− 0.30 ± 0.58	0.019^ƚ^
BuR	− 0.29 ± 0.54	0.008^ƚ^	− 0.34 ± 0.59	0.010^ƚ^
*Perioral soft tissues*				
TN	0.09 ± 0.26	0.069	− 0.03 ± 0.07	0.046^ƚ^
Sn	0.31 ± 0.46	0.005^ƚ,ǂ^	0.07 ± 0.54	0.528
A′	0.21 ± 0.64	0.089	− 1.03 ± 0.59	0.000^ƚ^
B′	0.28 ± 1.07	0.219^ǂ^	− 0.98 ± 0.99	0.000^ƚ^
Pog′	− 0.40 ± 0.89	0.024^ƚ^	− 0.24 ± 0.87	0.186
Me′	0.03 ± 0.81	0.822	0.15 ± 0.88	0.123^ǂ^

Values are all expressed as the mean ± SD.

*M*, mean; *SD*, standard deviation.

^ƚ^Significant difference of landmarks in the non-extraction and extraction groups, *P* < 0.05; *The data conformed to a normal distribution and a one-sample *t*-test was employed; ^ǂ^The data did not conform to normal distribution and a one-sample Wilcoxon’s test was used

**Table 4:** Comparison of changes between non-extraction and extraction groups*

Measurement	*t*-value	Z-value	*P*-value
*Nasolabial folds*			
NLF1L	− 3.771		0.000^ƚ^
NLF2L	− 5.169		0.000^ƚ^
NLF3L	− 5.216		0.000^ƚ^
L′	− 4.602		0.000^ƚ^
L′′	− 5.741		0.000^ƚ^
NLF1R		− 3.396	0.001^ƚ,ǂ^
NLF2R	− 4.506		0.000^ƚ,^
NLF3R	− 4.757		0.000^ƚ^
R′	− 3.277		0.002^ƚ^
R′′	− 4.516		0.000^ƚ,^
*Lips*			
ChL		− 3.928	0.000^ƚ,ǂ^
ChR		− 4.038	0.000^ƚ,ǂ^
ULP	− 5.721		0.000^ƚ^
LLP		− 4.883	0.000^ƚ,ǂ^
Ls		− 3.745	0.000^ƚ,ǂ^
Li		− 4.112	0.000^ƚ,ǂ^
CphL		− 3.506	0.000^ƚ,ǂ^
CphR		− 3.763	0.000^ƚ,ǂ^
STM		− 5.507	0.000^ƚ,ǂ^
*Cheeks*			
CkL		− 1.927	0.054^ǂ^
CkR		− 1.358	0.174^ǂ^
BuL	− 0.536		0.595
BuR	− 0.285		0.777
*Perioral soft tissues*			
TN		− 1.927	0.054^ǂ^
A′	− 7.261		0.000^ƚ^
B′		− 3.708	0.000^ƚ,ǂ^
Pog′	0.663		0.510
Sn		− 1.781	0.075^ǂ^
Me′		− 0.899	0.368^ǂ^

^ƚ^Significant difference between the non-extraction and extraction groups, *P* < 0.05; *If the data conformed to normal distribution and variances were uniform, an independent sample *t*-test was used; ^ǂ^If the data did not conform to a normal distribution and uniform variances, a Mann-Whitney U test was used

**Table 5:** Variation differences between different landmarks*

Measurement	Non-extraction	Extraction
	*P*-value	*P*-value
NLF1L–NLF2L	0.355	0.011^ƚ^
NLF1R–NLF2R	0.636	0.982^ǂ^
NLF1L–NLF1R	0.802^ǂ^	0.844
NLF2L–NLF2R	0.982	0.942
NLF3L–NLF3R	0.415	0.581
L′–L′′	0.995	1.000^ǂ^
R′–R′′	0.516	0.103
L′–R′	0.499	0.877
L′′–R′′	0.990	0.882^ǂ^
ChL–ChR	0.933	0.596
ULP–LLP	0.432	0.000^ƚ^
Ls–Li	0.068	0.057
CphL–CphR	0.248	0.800
CkL–CkR	0.904	0.604
BuL–BuR	0.455	0.701
A′–B′	0.777	0.946^ǂ^

^ƚ^Significant difference between the different landmarks, *P* < 0.05; ^*^The data conformed to normal distribution and variances uniform, and a paired samples *t*-test was used; ^ǂ^The data did not conform to a normal distribution and uniform variances, therefore a Wilcoxon signed-rank test was employed

In the analysis of changes in the lip region, all measures were negative in the extraction group (*P* < 0.05), and the negative change in lower lip (LLP) was greater than upper lip (ULP) (*P* < 0.001; ULP = − 0.93 mm, LLP = − 1.46 mm), indicating that orthodontic extraction had a greater effect on the LLP. ULP, labrale superior (Ls), labrale inferior (Li), crista philtri left (CphL), crista philtri right (CphR) and stomion (STM) showed positive changes in the non-extraction group (*P* < 0.05), which indicates that these soft tissues protrude outward, suggesting a positive effect of non-extraction orthodontics on some parts of the lips. In all measurement items of the lips, the changes of the extraction group were significantly different from those of the non-extraction group (*P* < 0.001), and there was a significant negative change in the extraction group, suggesting that orthodontic extraction may lead to flatter lips and orthodontic non-extraction may lead to more protruding lips.

In the soft tissue changes of cheek area, buccal left (BuL) and buccal right (BuR) of the extraction group and the non-extraction group were all negatively changed (*P* < 0.05), which turned into inward depression, and there was no significant difference between BuL and BuR changes in the extraction group or the non-extraction group, indicating that orthodontic treatment will cause inward concavity of soft tissues in some areas of the cheek regardless of extraction.

In the changes of perioral soft tissues, tip of nose (TN), soft-tissue A-point (A′) and soft-tissue B-point (B′) were a negative change in the extraction group (*P* < 0.05), however, in the non-extraction group, subnasale (Sn) was a positive change (*P* < 0.05) and soft-tissue pogonion (Pog′) was a negative change (*P* < 0.05), indicating that the changes in perioral soft tissues due to extraction and non-extraction orthodontics were inconsistent.

## Discussion

Due to the few anatomical markers and different types of the NLFs, the changes after orthodontic treatment are not as obvious as those in the lips and chin, so there have been few studies carried out on the relationship between the NLFs and orthodontics. In recent years, the emergence of new equipment and technologies has made it possible to study the NLFs. The aims of the present study were to investigate any potential changes in the NLFs and associated peripheral soft tissues after orthodontic treatment of female adults using 3dMD stereo tomography system.

Ahn et al. [17] found that after orthodontic extraction, the soft tissue in the perioral area was significantly changed in the sagittal direction, including the NLFs area, but no definite conclusion was reached. The present study found characteristic changes in the NLFs region after orthodontic treatment. No matter tooth extraction or not, NLFs had retraction trend after orthodontic treatment. One of the possible reasons is that during orthodontic treatment, due to the wearing of the appliance, masticatory muscles are used less frequently than when there is no orthodontic treatment. The change in the middle part of the nasolabial fold was greater than that in the upper part in the extraction group. The upper part of the nasolabial fold was less changed, probably because it was close to the nasal base, with less muscle attachment and less mobility, so it did not change much after the orthodontic treatment. There was no significant difference in the retraction of the left and right NLFs in the extraction and non-extraction groups. Baek et al. [18] used CBCT to study the three-dimensional morphological changes of the NLFs in 39 patients aged 18–40 years after orthodontic treatment. The results showed that retraction of the anterior teeth resulted in more pronounced changes in the NLFs in extraction patients under the age of 30 years compared to the non-extraction group, findings consistent with our results.

The present study found that no matter whether a tooth was extracted, the NLFs had a negative trend after orthodontic treatment, and it was more obvious in the extraction group than in the non-extraction group, which may be due to the retraction of anterior teeth in the extraction group. In clinical practice, we often find that young adult female patients’ faces become more harmonized after tooth extraction. Visually, for adult female patients younger than 30 years of age, the NLFs improved as the ULP tucks in, making them more youthful and beautiful [18]. Probably because of changes in skin elasticity and a reduction in soft tissue thickness and redistribution of subcutaneous adipose tissue with aging. The NLFs may become more visually apparent in older women after orthodontic treatment for tooth extraction [19, 20]. Therefore, in orthodontic treatment, tooth extraction should be considered more prudent for some adult female patients. In addition, the amount of anterior teeth retraction should be accurately controlled during extraction orthodontic treatment to prevent the NLFs from becoming too obvious and destroying the facial aesthetics.

Changes of the lips in the extraction group were showing inward depression, and the changing trend of the lips was LLP > ULP (*P* < 0.001; ULP = − 0.93 mm, LLP = − 1.46 mm). One possible reason is that the ULP was mainly moved inward with retraction of the upper incisors, and the change of the LLP was affected by the retraction of both upper and lower incisors; thus the LLP was retracted to a greater extent than the retraction of the ULP.

There were significant differences in the changes to all landmarks in the lips between the extraction and non-extraction groups. The extraction group showed a negative change, which was inwardly concave, and the non-extraction group showed a positive change, which was outwardly protruding. It was demonstrated that orthodontic extraction to retract the anterior teeth would improve the slightly convex patient’s profile aesthetics, and that orthodontic non-extraction treatment might make the lips more prominent, with the associated risk of damaging facial aesthetics.

In the present study, it was found that the landmarks on the left and right corners of the mouth in the tooth extraction group had a negative trend, while the measurement results in the non-extraction group were just the opposite, indicating that tooth extraction had a significant impact on the left and right mouth corners. After orthodontic treatment in the non-extraction group, the left and right corners of the mouth showed a positive trend, showing outward protrusion (cheilion left (ChL) = 0.42 mm, cheilion right (ChR) = 0.42 mm). In the extraction group, the negative change in the bilateral mouth corners was the largest among landmarks of the lips (*P* < 0.001; ChL = − 1.48 mm, ChR = − 1.43 mm), indicating that after the anterior teeth were retracted, the corners of the mouth located between the 3rd and 4th maxillary teeth also had a significant retracted trend.

Ashley [21] studies have shown that with age, the thickness of the soft tissue in the cheeks of adult women decreased. Our study found that after orthodontic treatment, both BuL and BuR were inwardly concave (*P* < 0.05) and that there was no significant difference in the changes of BuL and BuR between the tooth extraction and the non-extraction groups, indicating that whether or not the tooth was extracted, orthodontic treatment made the buccal region thinner, which might make the face look haggard and aged. For adult women seeking orthodontic treatment, doctors should inform patients in advance of possible changes in the soft tissues of the buccal region.

In the extraction group in this study, Pog′ did not change much, which might be due to the difference in the tension of the mentalis muscle before and after orthodontic treatment, the retraction of the lower anterior teeth, and/or the changes in the position of the condyle or mandible.

Based on the 3dMD stereo tomography system, this study applied a quantitative soft tissue three-dimensional analysis method, and preliminarily confirmed the characteristic change trend of the soft tissue around the nasolabial sulcus after tooth extraction and non-extraction orthodontics in female adults. Compared with non-extraction orthodontics, the negative change trend of NLFs in extraction orthodontics was clearly obvious. Whether different orthodontic treatment factors affect the soft tissue around the NLFs remains to be further explored. In clinical practice, orthodontists should pay attention to informing patients that the soft tissue at the NLFs may be sunken during the orthodontic process. This phenomenon has certain facial aesthetic risks for some adult female patients. When making a treatment plan to decide whether to extract a tooth or not, comprehensive factors such as the patient’s profile, oral condition, age, gender and race should be considered and actively communicated to the patient. The question of whether orthodontic extraction treatment should be performed in some female patients should be treated with a high degree of caution. At the same time, it is necessary to use various types of anchorage reasonably so that the amount of anterior teeth retraction in extraction orthodontic treatment can be more accurately controlled to prevent the NLFs from becoming too obvious and destroying facial aesthetics.

In recent years, the preferred method of facial soft tissue assessment has been increasingly three-dimensional measurement, including three-dimensional optical scanning technology and 3dMD stereo tomography. 3D optical scanning technology does not expose the patient to radiation, but due to its low accuracy (0.5 mm), the measurement data error is too large and cannot accurately truly reflect facial changes, so its application is therefore limited [22, 23]. 3dMD has no exposure of patients to radioactivity concerns and its measurement speed, accuracy and repeatability have been greatly improved. It supports quantitative measurement analysis, which meet the requirements of oral and maxillofacial accuracy, and is very convenient for the clinical recording soft tissue images of patients. Studies have shown that there is no significant difference between the results of profile soft tissue analysis using 3dMD and traditional photographic techniques. Therefore, in this study, the 3dMD stereo tomography system was used to obtain three-dimensional data and images of patients facial soft tissues.

There were some limitations to our study. First, the sample size was not enough to be reliably divided into a number of subgroups by age, and subsequent studies should therefore involve a larger cohort of patients. With a larger sample size, it will be possible to analyze changes in facial soft tissues after orthodontic treatment in patients of different ages. Second, there was a lack of male patients, and additional males should be enrolled in a future study to compare soft tissue changes between men and women. Third, the severity of NLFs varies among different skeletal facial types, and the changes in the NLFs after orthodontic treatment in patients with different skeletal facial types are not necessarily consistent. Fourth, the sample size should be increased in future studies to permit reliable comparisons in nasolabial fold changes in different skeletal facial types. Finally, although the present study required patients to relax without micro-expression when acquiring 3dMD facial images, it was still impossible to ensure that the subjects’ facial relaxation was consistent when acquiring 3dMD images at T0 and T1. Therefore, we also need to find a more accurate way to acquire facial information to reduce experimental errors.

## Conclusion

Both extractive and non-extractive orthodontics can cause depression of the NLFs, and the effects of extractive orthodontics were more obvious, thus carrying some aesthetic risks for particular adult female patients. Extractive orthodontic treatment can make the lips project inwards and improve facial aesthetics. However, non-extractive orthodontic treatment may make the mouth more convex and is also accompanied by some aesthetic risks.

When developing orthodontic treatment plans for female patients concerned about facial aesthetics, the decision to extract teeth should be made very carefully, and the amount of anterior teeth retraction should be more accurately controlled.

## Data Availability

The data of the current study are available from the corresponding author on reasonable request.

## Abbreviations

A′: Soft-tissue A-point
B′: Soft-tissue B-point
BuL: Buccal left
BuR: Buccal right
CBCT: Cone-beam computed tomography
ChL: Cheilion left
ChR: Cheilion right
CkL: Cheek left
CkR: Cheek right
CphL: Crista philtri left
CphR: Crista philtri right
L′: Outside of the left nasolabial folds
L″: Inside of the left nasolabial folds
Li: Labrale inferior
LLP: Lower lip
Ls: Labrale superior
Me′: Soft-tissue menton
NLFs: Nasolabial folds
NLF1L: The left upper nasolabial folds
NLF2L: The left middle nasolabial folds
NLF3L: The left lower nasolabial folds
NLF1R: The right upper nasolabial folds
NLF2R: The right middle nasolabial folds
NLF3R: The right lower nasolabial folds
Pog′: Soft-tissue pogonion
R′: Outside of the right nasolabial folds
R″: Inside of the right nasolabial folds
Sn: Subnasale
STM: Stomion
TN: Tip of nose
TPA: Transpalatal arch
T0: Orthodontic patients had 3dMD facial images taken before treatment
T1: After bracket removal at the end of treatment
ULP: Upper lip
